# Child Mortality in England During the First 2 Years of the COVID-19 Pandemic

**DOI:** 10.1001/jamanetworkopen.2022.49191

**Published:** 2023-01-09

**Authors:** David Odd, Sylvia Stoianova, Tom Williams, Peter Fleming, Karen Luyt

**Affiliations:** 1 School of Medicine, Division of Population Medicine, Cardiff University, United Kingdom; 2 National Child Mortality Database, Bristol Medical School, University of Bristol, St Michael’s Hospital, Bristol, United Kingdom; 3 Centre for Academic Child Health, Population Health Sciences, Bristol Medical School, University of Bristol, United Kingdom

## Abstract

**Importance:**

During the first year of the COVID-19 pandemic, child mortality in England was the lowest on record, but if this trend will continue, or if unrecognized morbidity during the first year of the pandemic will manifest as increased deaths over the next few years is unclear.

**Objective:**

To examine the risks and patterns of childhood deaths before and during the COVID-19 pandemic.

**Design, Setting, and Participants:**

This population-based cohort study includes all child deaths in England from April 1, 2019, to March 31, 2022.

**Exposures:**

The year of death.

**Main Outcomes and Measures:**

The primary outcome measure is risk of death.

**Results:**

Of the 9983 child deaths reported during the study period, 9872 (98.8%) were linked to demographic and population data with 3409 deaths (34.5%) between April 2019 and March 2020, 3035 (30.7%) between April 2020 and March 2021, and 3428 (34.7%) between April 2021 and March 2022. Most deaths occurred in children who were younger than 1 year (6257 of 9872 [62.7%]), the majority were male (5534 of 9760 [56.7%]), and lived in an urban area (8766 of 9872 [88.8%]). The risk of death was lower between April 2020 and March 2021 (relative risk [RR], 0.89 [95% CI, 0.84-0.93]), but not between April 2021 and March 2022 (RR, 1.00 [95% CI, 0.95-1.05]) when compared with April 2019 to March 2020. A population attributable risk (PAF) of 4.0% (95% CI, 0.1%-6.8%) suggested fewer deaths occurred during the whole 3-year period than expected. Reductions were seen in risk of dying by infection (PAF, 22.8% [95% CI, 8.2%-37.0%]) and underlying disease (PAF, 13.3% [95% CI, 8.1%-18.8%]), but there was evidence of an increasing risk of death by trauma (PAF, 14.7% [95% CI, 2.9%-25.2%]). Any reduction in the risk of death was greater in rural areas than in urban areas (RR, 0.73 [95% CI, 0.63-0.85] vs RR, 0.91 [95% CI, 0.86-0.95]) and was not seen in children older than 9 years.

**Conclusions and Relevance:**

In this cohort study, there was a significant reduction in all-cause child mortality during the first year of the COVID-19 pandemic (2020-2021), which returned to close to prepandemic levels the following year (2021-2022). However, there was a net reduction in deaths despite this, with 4% fewer deaths during the 3-year period than would have been expected from the 2019 to 2020 risks. The reductions were largest in rural areas and in children younger than 10 years.

## Key Points

**Question:**

What were the risks and patterns of childhood deaths before and during the COVID-19 pandemic?

**Findings:**

In this cohort study, there were 3409 childhood deaths from April 2019 to March 2020, 3035 deaths from April 2020 to March 2021, and 3428 deaths from April 2021 to March 2022. Overall risk of death was significantly lower from 2020 to 2021, but not from 2021 to 2022 when compared with the reference year of 2019 to 2020.

**Meaning:**

These findings suggest that there was a significant reduction in all-cause child mortality during the first year of the COVID-19 pandemic (2020-2021), which returned to near prepandemic levels the following year (2021-2022).

## Teaser

This cohort study examines the risks and patterns of childhood deaths in England before and during the COVID-19 pandemic.

## Introduction

Since the start of the COVID-19 pandemic there have been over 100 000 excess deaths in England and Wales^1^ and 14.9 million worldwide.^2^ While we know the case fatality rate of COVID-19 in children is low, the impact of widespread changes to health care and society during the last 2 years have had broad impacts on child health and access to services. However, for the first year of the pandemic, child mortality in England was likely the lowest on record, with dramatic reductions in infections and deaths from underlying conditions.^3^ However, while overall child mortality fell, concerns remain in some categories (eg, suicide^4^) and urban communities. In addition, it is unclear, as levels of COVID-19 infection fall, and services return to normal, if this trend will continue, or if unrecognized morbidity during the first year of the pandemic (eg, later diagnosis of malignancies) will manifest as increased deaths during the next few years.

In England, all deaths of children are reviewed by Child Death Overview Panels (CDOPs), and data collated by the National Child Mortality Database (NCMD), with death notifications required by statute within 48 hours. The aim of this analysis was to quantify the relative risk of childhood deaths, across the whole of England, before and during the first 2 years of the COVID-19 pandemic, and identify any changes that may have occurred, in patterns of mortality during this period.

## Methods

This cohort study followed the Strengthening the Reporting of Observational Studies in Epidemiology (STROBE) reporting guideline. The Chair of the Central Bristol National Health Service (NHS) research ethics committee reviewed this work and confirmed that NHS ethical approval or individual consent was not required. NCMD commenced data collection on April 1, 2019, and collected data from all Child Death Overview Panels (CDOPs) across England.^5^ Parent and public involvement guided the design and setting up of the NCMD at establishment and real-time child mortality surveillance system at the beginning of the COVID-19 pandemic.

In the initial notification to NCMD, the CDOPs reported baseline characteristics of the child, from which the following data were derived: (1) sex of individual (female, male, other [including not known]); (2) ethnic group derived from self-reported health records (ie, Asian or Asian British, Black or Black British, mixed, other [Arab or any other ethnic group], unknown, and White); (3) age at death; and (4) from the child’s home postcode, a measure of deprivation, the rural or urban nature of the area, and the region of England where the child lived.

From March 1, 2020, linkage with virology polymerase chain reaction results was performed with Public Health England and from April 2020, the Joint Agency Response to unexpected child deaths was amended to include postmortem SARS-CoV-2 viral swabs from all children dying with no immediately identifiable cause.^6^ In this work, similar to our previous work,^7^ in order to obtain a provisional category of death, all child deaths reported to NCMD were coded by 3 independent coders (all pediatricians) to identify the likely category of the cause of death. Coders recorded a provisional category of death or that there was insufficient information. If 2 or more coders agreed on a category, this was taken as the most likely category. Where no 2 coders agreed, the category highest in the following hierarchy was used (based on categorization used by CDOPs),^8^ including (1) suicide; (2) substance abuse; (3) trauma; (4) malignant neoplasm; (5) underlying medical condition; (6) intrapartum event; (7) preterm birth; (8) infection; or (9) sudden unexpected death in infancy or childhood (SUDIC).

The underlying population profile was obtained from the 2019 and 2020 Office of National Statistics (ONS) population estimates^9^ for all measures except ethnicity. The population profile from 2021 to 2022 was derived from 2020 data, assuming no net migration, and using birth data from 2020 to derive the population for those younger than 1 year. Measures of rural or urban status and deprivation were derived at the level of the lower layer super output areas (LSOA). Deprivation was derived from the ONS index of multiple deprivation (IMD)^10^; calculated from a number of measures (eg, employment, crime, etc) across the local area. The population split into 10 equal sized (by population) deciles with a lower value suggesting greater deprivation. Population ethnicity profile was based on the 2011 census data for all years^11^ at the middle layer super output area (MSOA) level; this was amended using the estimated population changes to reflect likely current population structures in 2019^12^ by modifying the populations of each ethnic group by the predicted demographic changes. LSOAs and MSOAs are derived at a population of around 1500 and 7200 people, respectively.

### Statistical Analysis

Deaths of children occurring from April 1, 2019, until March 31, 2022, were identified and divided into three 12-month periods from April to March (ie, 2019-2020, 2020-2021, and 2021-2022). Initially, we compared the characteristics of those children who died across the 3 time periods and the causes of death seen. Comparisons were made using χ^2^ for categorical data.

We then calculated the estimated risk (per 100 000 person-years) for the 3 time periods, and the relative risk compared with the 2019 to 2020 time period (ie, prepandemic). The probability that the risk changed during the 3 years, and the population attributable risk fraction (deriving the association if risk across the 3 years had remained at the 2019 to 2020 levels) were also derived; and we then repeated the main model restricting it to each provisional category of death.

Next, we stratified the association by year and for different demographic groups (age of death [less than 1 year, 1-4 years, 5-9 years, 10-14 years, or 15-17 years], ethnic group, sex, region of the country, area, and deprivation score). Finally, because of clear age-dependent changes in COVID-19 mortality seen in previous work,^3,7,13^ we tested to see if the changes in the risk of death, for each cause of death, varied by the age category of the children; it was not performed for age-dependent conditions, such as intrapartum and preterm death. In addition, due to small numbers, analysis was restricted to children older than 9 years for deaths by suicide and substance abuse. The model was tested using the likelihood ratio test to see if the profile of risk seen across the 3 years differed by the patient characteristics or categories of death.

Data are presented as median number (%) or relative risk ratio (RR) (95% CI). Where frequency counts were below 5, or could be derived, absolute numbers are not presented. Analysis was performed using Stata version 17 (StataCorp). Comparisons were made using the χ^2^ or likelihood ratio test as appropriate, and *P* values <.05 were considered evidence of statistical significance.

## Results

In total, there were 9983 deaths within the study period reported to NCMD, of which 9872 (98.8%) were linked to demographic and population data (3409 [34.5%] deaths in 2019-2020, 3035 [30.7%] in 2020-2021, and 3428 [34.7%] in 2021-2022) (Figure and Table 1). The age profile of child deaths in England during the 3 years varied, with a lower proportion of deaths from 2020 to 2021 between children aged 1 to 4 years and 5 to 9 years (263 [8.7%] and 195 [6.4%], respectively) than in 2019 to 2020 (393 [11.5%] and 244 [7.2%], respectively), and 2021 to 2022 (359 [10.5%] and 240 [7.0%], respectively). From 2020 to 2021, a smaller proportion of child deaths occurred in rural areas (299 [9.9%]), compared with 2019 to 2020 (407 [11.9%]) and 2021 to 2022 (400 [11.7%]) (*P* = .02). The cause of death also appeared to vary across the 3 years studied (eTable in the Supplement); with lower proportions of death by infection (82 [2.8%]) and underlying disease (807 [27.4%]) from the 2020 to 2021 period than the 2019 to 2020 period (165 [5.0%] and 1080 [32.8%], respectively) and the 2021 to 2022 period (161 [4.8%] and 983 [29.1%], respectively) (*P* < .001).

**Figure.**
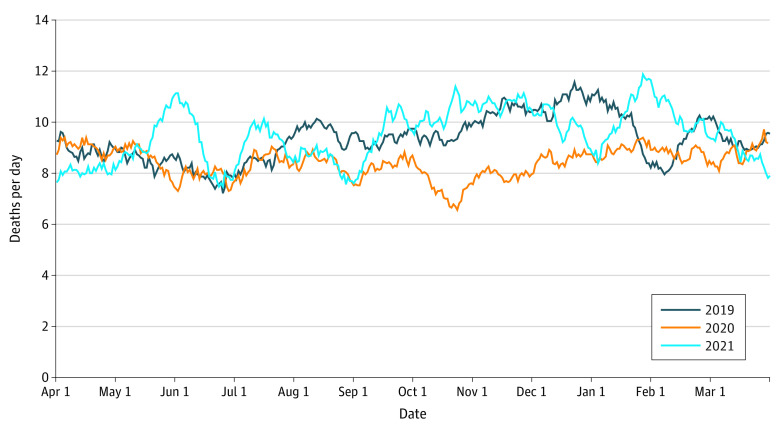
Number of Deaths per Day, Across the 3 Years Between 2019 to 2020, 2020 to 2021, and 2021 to 2022 (April to March)

**Table 1.** Characteristics of the Populations of Child Deaths Reported to NCMD in England Between April 2019 and March 2022

Measure	Total No.	Child deaths reported, No. (%)			*P* value
		2019-2020	2020-2021	2021-2022	
All deaths	9872	3409	3035	3428	
Age of death, y	9872				
<1		2150 (63.1)	1973 (65.0)	2134 (62.3)	.007
1-4		393 (11.5)	263 (8.7)	359 (10.5)	
5-9		244 (7.2)	195 (6.4)	240 (7.0)	
10-14		286 (8.4)	266 (8.8)	309 (9.0)	
15-17		336 (9.9)	338 (11.1)	386 (11.3)	
Sex	9760				
Female		1475 (43.6)	1297 (43.4)	1454 (42.9)	.85
Male		1909 (56.4)	1691 (56.6)	1934 (57.1)	
Area of residence	9872				
Urban		3002 (88.1)	2736 (90.2)	3028 (88.3)	.02
Rural		407 (11.9)	299 (9.9)	400 (11.7)	
Ethnicity	9128				
Asian or British Asian		581 (18.9)	478 (17.3)	599 (18.2)	.38
Black or British Black		262 (8.5)	253 (9.2)	273 (8.3)	
Mixed		202 (6.6)	164 (5.9)	227 (6.9)	
Other^a^		88 (2.9)	63 (2.3)	84 (2.6)	
White		1937 (63.1)	1801 (65.3)	2116 (64.1)	
Region of residence	9872				
East Midlands		285 (8.4)	233 (7.7)	289 (8.4)	.64
East of England		334 (9.8)	276 (9.1)	336 (9.8)	
London		600 (17.6)	552 (18.2)	533 (15.6)	
North East		151 (4.4)	130 (4.3)	170 (5.0)	
North West		488 (14.3)	439 (14.5)	512 (14.9)	
South East		469 (13.8)	407 (13.4)	474 (13.8)	
South West		250 (7.3)	232 (7.6)	248 (7.2)	
West Midlands		466 (13.7)	432 (14.2)	487 (14.2)	
Yorkshire and the Humber		366 (10.7)	334 (11.0)	379 (11.1)	
Deprivation decile	9872				
1/2 (most deprived)		1153 (33.8)	1035 (34.1)	1163 (33.9)	.97
3/4		776 (22.8)	699 (23.0)	773 (22.6)	
5/6		625 (18.3)	540 (17.8)	583 (17.0)	
7/8		483 (14.2)	411 (13.5)	494 (14.4)	
9/10 (least deprived)		372 (10.9)	350 (11.5)	415 (12.1)	
COVID-19 positive within 28 d of death	9872	NA	40 (1.3)	101 (3.0)	<.001

Abbreviation: NA, not applicable.

^a^ Other ethnic group (Arab, any other ethnic group).

Overall risk of death was lower from 2020 to 2021 (RR, 0.89 [95% CI, 0.84-0.93]) but not in 2021 to 2022 (RR, 1.00 [95% CI, 0.95-1.05]) when compared with the reference year of 2019 to 2020 (Table 2). However, there is still evidence that 4.0% (95% CI, 0.1%-6.8%) fewer deaths occurred during the whole 3-year period than would have been expected if risk had remained at the 2019 to 2020 levels throughout. When stratifying by cause of death, a similar profile was seen with infection (2020-2021: RR, 0.50 [95% CI, 0.38-0.64] and 2021-2022: RR, 0.97 [95% CI, 0.78-1.21]; *P* < .001) and underlying disease (2020-2021: RR, 0.74 [95% CI, 0.68-0.81] and 2021-2022: RR, 0.91 [95% CI, 0.83-0.99]; *P* < .001). In contrast, there was evidence of an increasing risk of death by trauma (2020-2021: RR, 1.20 [95% CI, 0.97-1.47] and 2021-2022: RR, 1.32 [95% CI, 1.08-1.62]; *P* = .03) and SUDIC across the 3 years (2020-2021: RR, 0.96 [95% CI, 0.84-1.10] and 2021-2022: RR, 1.13 [95% CI, 0.99-1.28]; *P* = .04), although not all results were statistically significant. There was no difference in the overall risk of death across the 3 years for malignant neoplasm (2020-2021: RR, 1.00 [95% CI, 0.84-1.19] and 2021-2022: RR, 0.96 [95% CI, 0.81-1.14]; *P* = .88), preterm birth (2020-2021: RR, 0.96 [95% CI, 0.88-1.05] and 2021-2022: RR, 1.06 [95% CI, 0.97-1.16]; *P* = .11), intrapartum events (2020-2021: RR, 1.13 [95% CI, 0.91-1.39] and 2021-2022: RR, 1.10 [95% CI, 0.89-1.36]; *P* = .50), substance abuse (2020-2021: RR, 0.50 [95% CI, 0.23-1.06] and 2021-2022: RR, 0.45 [95% CI, 0.20-0.98]; *P* = .07), or suicide (2020-2021: RR, 1.09 [95% CI, 0.84-1.41] and 2021-2022: RR, 1.21 [95% CI, 0.94-1.56]; *P* = .32).

**Table 2.** Risk (per 100 000 Children) by Year of Death, Stratified by Cause of Death

Measure	No.	Risk (per 100 000 population)			Relative risk (vs 2019-2020)				
		2019-2020	2020-2021	2021-2022	2019-2020	2020-2021	2021-2022	Population attributable risk fraction (95% CI)	*P* value
All deaths	9872	28.3 (27.4 to 29.3)	25.1 (24.2 to 26.0)	28.3 (27.3 to 29.2)	1 [Reference]	0.89 (0.84 to 0.93)	1.00 (0.95 to 1.05)	–4.00 (–6.8 to –0.1)	<.001
Death by cause^a^									
Malignant neoplasm	760	2.1 (1.9 to 2.4)	2.1 (1.9 to 2.4)	2.0 (1.8 to 2.3)	1 [Reference]	1.00 (0.84 to 1.19)	0.96 (0.81 to 1.14)	–1.4 (–12.0 to 8.1)	.88
Preterm birth	2738	7.5 (7.0 to 8.0)	7.2 (6.7 to 7.7)	8.0 (7.5 to 8.5)	1 [Reference]	0.96 (0.88 to 1.05)	1.06 (0.97 to 1.16)	0.7 (–4.7 to 5.9)	.11
Intrapartum event	538	1.4 (1.2 to 1.6)	1.6 (1.3 to 1.8)	1.5 (1.3 to 1.8)	1 [Reference]	1.13 (0.91 to 1.39)	1.10 (0.89 to 1.36)	7.1 (–5.4 to 18.1)	.50
Infection	408	1.4 (1.2 to 1.6)	0.7 (0.5 to 0.8)	1.3 (1.1 to 1.6)	1 [Reference]	0.50 (0.38 to 0.64)	0.97 (0.78 to 1.21)	–22.8 (–37.0 to –8.2)	<.001
Trauma	572	1.3 (1.1 to 1.6)	1.6 (1.4 to 1.9)	1.8 (1.5 to 2.0)	1 [Reference]	1.20 (0.97 to 1.47)	1.32 (1.08 to 1.62)	14.7 (2.9 to 25.2)	.03
Substance abuse	39	0.2 (0.1 to 0.3)	0.1 (0.0 to 0.2)	0.1 (0.0 to 0.1)	1 [Reference]	0.50 (0.23 to 1.06)	0.45 (0.20 to 0.98)	–54.4 (–100.0 to –13.7)	.07
Suicide	361	0.9 (0.71.1)	1.0 (0.8 to 1.2)	1.1 (0.9 to 1.3)	1 [Reference]	1.09 (0.84 to 1.41)	1.21 (0.94 to 1.56)	9.1 (–6.3 to 22.2)	.32
SUDIC	1340	3.6 (3.3 to 4.0)	3.4 (3.1 to 3.8)	4.1 (3.7 to 4.4)	1 [Reference]	0.96 (0.84 to 1.10)	1.13 (0.99 to 1.28)	2.7 (–5.1 to 10.0)	.04
Underlying disease	2870	9.0 (8.5 to 9.6)	6.7 (6.2 to 7.1)	8.1 (7.6 to 8.7)	1 [Reference]	0.74 (0.68 to 0.81)	0.91 (0.83 to 0.99)	–13.3 (–18.8 to –8.1)	<.001

Abbreviation: SUDIC, sudden unexpected death in infancy or childhood.

^a^ 9626 included in deaths by cause.

Compatible with the univariable analysis, the reduction in the risk of death from 2020 to 2021 appeared greater in rural areas than in urban areas (RR, 0.73 [95% CI, 0.63-0.85] vs RR, 0.91 [95% CI, 0.86-0.95]) when compared with 2019 to 2020, but both estimates returned to prepandemic levels in the 2021 to 2022 period (RR, 0.99 [95% CI, 0.86-1.13] vs RR, 1.00 [95% CI, 0.95-1.05]; *P* value for the interaction = .009) (Table 3). Similarly, any reduction in the risk of death during 2020 to 2021 appeared to differ by the age of the child (*P* value for the interaction = .01), with bigger reductions in children aged 1 to 4 years and 5 to 9 years (RR, 0.68 [95% CI, 0.58-0.80] and RR, 0.80 [95% CI, 0.66-0.96], respectively) than in other age groups (<1 year: RR, 0.94 [95% CI, 0.89-1.00]; 10-14 years: RR, 1.04 [95% CI, 0.88-1.22]; 15-17 years: RR, 1.10 [95% CI, 0.94-1.26]). There was little evidence that the overall changes in risk seen across the 3 years varied by ethnicity (*P* value for the interaction = .38), region of England (*P* value for the interaction = .52), or deprivation (*P* value for the interaction = .51).

**Table 3.** Risk by Year of Death, Stratified by Child Characteristics

Measure	No.	Risk per 100 000 population, 95% CI			Relative risk, 95% CI			
		2019-2020	2020-2021	2021-2022	2019-2020	2020/2021	2021/2022	*P* for interaction
Age of death, y								
<1	6256	346.2 (331.7-361.2)	326.6 (312.3-341.3)	353.7 (338.8-369.0)	1 [Reference]	0.94 (0.89-1.00)	1.02 (0.96-1.08)	.01
1-4	1015	14.7 (13.2-16.2)	10.0 (8.8-11.3)	14.1 (12.6-15.6)	1 [Reference]	0.68 (0.58-0.80)	0.96 (0.83-1.11)	
5-9	679	6.9 (6.1-7.8)	5.5 (4.8-6.3)	6.8 (6.0-7.7)	1 [Reference]	0.80 (0.66-0.96)	0.99 (0.83-1.18)	
10-14	861	8.5 (7.6-9.6)	7.7 (6.8-8.7)	8.9 (7.9-9.9)	1 [Reference]	0.91 (0.77-1.07)	1.04 (0.88-1.22)	
15-17	1060	18.3 (16.4-20.4)	18.8 (16.1-20.0)	20.0 (18.1-22.1)	1 [Reference]	0.98 (0.84-1.14)	1.10 (0.94-1.26)	
Sex								
Female	4226	25.2 (23.9-26.5)	22.0 (20.8-23.2)	24.7 (23.4-26.0)	1 [Reference]	0.87 (0.81-0.94)	0.98 (0.91-1.05)	.85
Male	5534	31.0 (29.6-32.4)	27.3 (26.03-28.6)	31.2 (29.8-32.6)	1 [Reference]	0.88 (0.82-0.94)	1.01 (0.95-1.07)	
Area of residence								
Urban	8766	29.5 (28.4-30.6)	26.7 (25.7-27.7)	29.5 (28.5-30.6)	1 [Reference]	0.91 (0.86-0.95)	1.00 (0.95-1.05)	.01
Rural	1106	22.0 (19.9-24.3)	16.1 (14.3-18.0)	21.8 (19.7-24.1)	1 [Reference]	0.73 (0.63-0.85)	0.99 (0.86-1.13)	
Ethnicity								
Asian or British Asian	1658	42.4 (39.0-46.0)	34.9 (31.8-38.2)	43.7 (40.3-47.4)	1 [Reference]	0.82 (0.73-0.93)	1.03 (0.92-1.16)	.38
Black or British Black	788	36.6 (32.3-41.3)	35.4 (31.1-40.0)	38.2 (33.8-43.0)	1 [Reference]	0.97 (0.81-1.15)	1.04 (0.88-1.23)	
Mixed	593	33.9 (29.4-38.9)	27.5 (23.5-32.1)	38.1 (33.3-43.4)	1 [Reference]	0.81 (0.66-1.00)	1.12 (0.93-1.38)	
Other^a^	235	22.0 (17.7-27.2)	15.8 (12.1-20.2)	21.0 (16.8-26.1)	1 [Reference]	0.72 (0.52-0.99)	0.95 (0.71-1.29)	
White	5854	21.5 (20.6-22.5)	20.0 (19.1-21.0)	23.5 (22.5-24.5)	1 [Reference]	0.93 (0.87-0.99)	1.09 (1.03-1.16)	
Region of residence								
East Midlands	807	28.4 (25.2-31.9)	23.4 (20.2-26.3)	28.8 (25.5-32.3)	1 [Reference]	0.82 (0.68-0.97)	1.01 (0.86-1.19)	.52
East of England	946	24.8 (22.2-27.6)	20.3 (18.0-22.9)	24.8 (22.2-27.6)	1 [Reference]	0.82 (0.69-0.96)	1.00 (0.86-1.16)	
London	1685	29.5 (27.2-32.0)	27.0 (24.8-29.3)	25.8 (23.46-28.1)	1 [Reference]	0.91 (0.81-1.03)	0.87 (0.77-0.98)	
North East	451	25.8 (23.6-28.1)	24.4 (20.3-28.9)	32.0 (27.4-37.2)	1 [Reference]	0.86 (0.68-1.09)	1.13 (0.91-1.40)	
North West	1439	31.2 (28.5-34.1)	27.9 (25.4-30.7)	32.6 (29.8-35.5)	1 [Reference]	0.89 (0.79-1.02)	1.04 (0.92-1.18)	
South East	1350	23.8 (21.7-26.1)	20.5 (18.6-22.6)	24.0 (21.9-26.3)	1 [Reference]	0.86 (0.75-0.98)	1.01 (0.89-1.15)	
South West	730	22.6 (19.9-25.5)	20.8 (18.2-23.7)	22.4 (19.7-25.4)	1 [Reference]	0.92 (0.77-1.10)	0.99 (0.83-1.18)	
West Midlands	1385	35.8 (32.7-39.2)	33.8 (32.7-39.2)	37.3 (34.1-40.8)	1 [Reference]	0.92 (0.81-1.05)	1.034 (0.92-1.18)	
Yorkshire and the Humber	1079	31.3 (28.2-34.6)	28.4 (25.5-31.7)	32.3 (29.2-35.8)	1 [Reference]	0.91 (0.78-1.05)	1.03 (0.90-1.20)	
Deprivation decile								
1/2 (Most deprived)	3351	40.2 (37.9-42.5)	35.9 (33.7-38.1)	40.1 (37.8-42.4)	1 [Reference]	0.89 (0.82-0.97)	1.00 (0.92-1.08)	.51
3/4	2248	31.2 (29.0-33.5)	27.9 (25.9-30.1)	30.7 (28.6-33.0)	1 [Reference]	0.89 (0.81-0.99)	0.99 (0.89-1.09)	
5/6	1748	27.5 (25.4-29.8)	23.6 (21.6-25.7)	25.5 (23.4-27.6)	1 [Reference]	0.86 (0.76-0.96)	0.92 (0.83-1.03)	
7/8	1388	22.3 (20.3-24.3)	18.8 (17.0-20.7)	22.7 (20.7-24.8)	1 [Reference]	0.84 (0.74-0.96)	1.02 (0.90-1.16)	
9/10 (Least deprived)	1137	16.7 (15.1-18.5)	15.7 (14.1-17.4)	18.9 (17.1-20.8)	1 [Reference]	0.94 (0.81-1.08)	1.13 0.98-1.30)	

^a^ Other ethnic group (Arab, any other ethnic group).

Finally, there was no evidence that the overall changes in risk of dying from malignant neoplasm (*P* value for the interaction = .24), trauma (*P* value for the interaction = .28), substance abuse (*P* value for the interaction = .92), suicide (*P* value for the interaction = .33) or underlying disease (*P* value for the interaction = .47) varied by the age category across the 3-year period (Table 4). However, risk of death by infections appeared to differ across the 3-year period, depending on the age of the child (*P* value for the interaction = .02). Children younger than 14 years had a lower risk of death from 2020 to 2021 compared with 2019 to 2020; with similar risks from 2021 to 2022 to those seen in the prepandemic year. In contrast, older children aged 15 to 17 years did not demonstrate a reduction in deaths from infection from 2020 to 2021 (RR, 1.27 [95% CI, 0.56-2.89]) and had a higher risk of death from 2021 to 2022 than in 2019 to 2020 (RR, 2.56 [95% CI, 1.24-5.29]). There also appeared to be a different profile of deaths from SUDIC across the 3 years, by age (*P* value for the interaction = .04), with a reduction in risk seen only in 1 group (age 1-4 years: RR, 0.56; [95% CI, 0.38-0.82]) during 2020 to 2021 compared with 2019 to 2020.

**Table 4.** Risk (per 100 000 Children) by Year of Death Stratified by Cause and Age of Death^a^

Measure	No.	Risk per 100 000 population, 95% CI			Relative risk, 95% CI			
		2019-2020	2020-2021	2021-2022	2019-2020	2020/2021	2021/2022	*P *for interaction
Malignant neoplasm, y								.24
<1 y	49	3.54 (2.22-5.36)	1.66 (0.79-3.04)	2.82 (1.64-4.51)	1 [Reference]	0.47 (0.22-0.99)	0.80 (0.42-1.50)	
1-4	167	2.13 (1.61-2.75)	2.05 (1.54-2.67)	2.20 (1.66-2.85)	1 [Reference]	0.96 (0.66-1.40)	1.03 (0.71-1.49)	
5-9	216	1.95 (1.52-2.47)	2.03 (1.59-2.56)	2.13 (1.68-2.67)	1 [Reference]	1.04 (0.75-1.45)	1.09 (0.79-1.52)	
10-14	183	1.97 (1.52-2.50)	2.01 (1.56-2.54)	1.38 (1.01-1.83)	1 [Reference]	1.02 (0.73-1.43)	0.70 (0.48-1.01)	
15-17	145	2.29 (1.65-3.10)	2.77 (2.07-3.63)	2.64 (1.97-3.47)	1 [Reference]	1.21 (0.80-1.81)	1.15 (0.77-1.73)	
Infection, y								.02
<1	160	9.34 (7.09-12.07)	6.29 (4.45-8.64)	10.61 (8.17-13.5)	1 [Reference]	0.67 (0.45-1.01)	1.14 (0.80-1.62)	
1-4	85	1.60 (1.16-2.16)	0.45 (0.24-0.79)	1.18 (0.80-1.68)	1 [Reference]	0.28 (0.15-0.54)	0.73 (0.46-1.17)	
5-9	60	0.90 (0.62-1.28)	0.28 (0.14-0.52)	0.51 (0.30-0.81)	1 [Reference]	0.31 (0.15-0.64)	0.57 (0.32-1.01)	
10-14	53	0.66 (0.41-0.99)	0.26 (0.12-0.50)	0.63 (0.40-0.96)	1 [Reference]	0.40 (0.18-0.87)	0.96 (0.53-1.74)	
15-17	50	0.55 (0.26-1.00)	0.69 (0.37-1.18)	1.40 (0.92-2.04)	1 [Reference]	1.27 (0.56-2.89)	2.56 (1.24-5.29)	
Trauma								.28
<1	72	2.58 (1.47-4.18)	5.30 (3.62-7.48)	3.98 (2.55-5.92)	1 [Reference]	2.06 (1.13-3.75)	1.54 (0.82-2.91)	
1-4	140	1.53 (1.10-2.07)	1.93 (1.44-2.54)	1.88 (1.39-2.50)	1 [Reference]	1.26 (0.84-1.91)	1.23 (0.82-1.87)	
5-9	44	0.23 (0.10-0.45)	0.51 (0.30-0.80)	0.51 (0.30-0.81)	1 [Reference]	2.25 (0.98-5.17)	2.26 (0.98-5.21)	
10-14	91	0.80 (0.53-1.17)	0.84 (0.57-1.21)	1.00 (0.70-1.40)	1 [Reference]	1.05 (0.62-1.77)	1.25 (0.75-2.06)	
15-17	225	3.82 (2.98-4.83)	3.46 (2.67-4.41)	4.66 (3.75-5.73)	1 [Reference]	0.91 (0.65-1.27)	1.22 (0.89-1.67)	
Substance abuse, y								.92
10-14	NR^b^	0.09 (0.02-0.26)	0.03 (0.00-0.16)	0.03 (0.00-0.16)	1 [Reference]	0.33 (0.03-3.13)	0.32 (0.03-3.08)	
15-17	NR^b^	0.93 (0.54-1.49)	0.48 (0.22-0.91)	0.41 (0.18-0.82)	1 [Reference]	0.52 (0.23-1.16)	0.44 (0.19-1.03)	
Suicide, y								.33
10-14	NR^b^	0.72 (0.46-1.06)	0.84 (0.57-1.21)	1.15 (0.82-1.56)	1 [Reference]	1.18 (0.69-2.03)	1.60 (0.97-2.66)	
15-17	NR^b^	4.59 (3.66-5.68)	4.74 (3.80-5.83)	4.82 (3.89-5.90)	1 [Reference]	1.03 (0.77-1.39)	1.05 (0.78-1.41)	
SUDIC, y								.04
<1	853	45.41 (40.26-51.03)	45.37 (40.16-51.08)	49.22 (43.78-55.15)	1 [Reference]	1.00 (0.85-1.18)	1.08 (0.92-1.28)	
1-4	194	2.76 (2.17-3.46)	1.55 (1.11-2.11)	3.10 (2.45-3.86)	1 [Reference]	0.56 (0.38-0.82)	1.12 (0.81-1.54)	
5-9	75	0.57 (0.35-0.87)	0.68 (0.43-1.01)	0.88 (0.60-1.25)	1 [Reference]	1.20 (0.66-2.17)	1.56 (0.89-2.74)	
10-14	108	0.83 (0.55-1.21)	1.08 (0.76-1.48)	1.23 (0.89-1.66)	1 [Reference]	1.29 (0.79-2.11)	1.48 (0.92-2.38)	
15-17	110	1.58 (1.06-2.27)	2.18 (1.57-2.96)	2.07 (1.48-2.82)	1 [Reference]	1.38 (0.86-2.22)	1.31 (0.81-2.11)	
Underlying disease, y								.47
<1	1703	102.58 (94.76-110.86)	83.29 (76.17-90.90)	93.31 (85.76-101.34)	1 [Reference]	0.81 (0.72-0.92)	0.91 (0.81-1.02)	
1-4	395	5.93 (5.04-6.93)	3.75 (3.05-4.57)	5.37 (4.51-6.35)	1 [Reference]	0.63 (0.49-0.81)	0.91 (0.72-1.14)	
5-9	266	2.97 (2.43-3.59)	1.86 (1.44-2.37)	2.70 (2.19-3.30)	1 [Reference]	0.63 (0.46-0.85)	0.91 (0.69-1.20)	
10-14	301	3.13 (2.56-3.79)	2.33 (1.85-2.90)	3.33 (2.75-3.99)	1 [Reference]	0.74 (0.56-0.99)	1.06 (0.82-1.38)	
15-17	205	4.04 (3.17-5.07)	3.14 (2.39-4.05)	3.73 (2.91-4.70)	1 [Reference]	0.78 (0.55-1.09)	0.92 (0.67-1.28)	

Abbreviation: SUDIC, sudden unexpected death in infancy or childhood.

^a^ Due to small numbers of deaths caused directly by preterm birth or intrapartum events after 1 year of age, these categories have not been reported.

^b^ In both the suicide and substance abuse categories, one age group had less than 5 children and the number of childer in the other age group was much higher. However, the smaller group would be derivable from data presented elsewhere in the study, thus both numbers were suppressed.

## Discussion

In this cohort study, the number of deaths of children in England dropped significantly during the first year of the pandemic (ie, 2020-2021) but returned to baseline levels in the following year (ie, 2020-2022). This fall was more pronounced in rural areas than urban areas. However, there was little to suggest an excess of deaths during the 2021 to 2022 period overall, or for most subgroups investigated. Disruption to health care services, and potentially later diagnoses or underdiagnosed conditions, appear not to have had a measurable impact on mortality. For most groups the benefits seen in 2020 to 2021 of a reduction in deaths from infectious agents has also disappeared (again without a rebound to high levels), but for the oldest children, where no initial benefit was seen, risks of death are now well above prepandemic levels. In contrast to most deaths, those from trauma have increased during the 3 years. This association does not appear to be restricted to a particular age group. Like the rest of this work, interpretation of underlying causal processes is difficult; but an underlying trend unrelated to COVID-19 may explain some of the associations seen.

While the risks of death may have returned to levels comparable with prepandemic rates, there still remains a cohort of approximately 350 children who are alive today having not died from infections because of the widespread reductions to infectious disease transmission consequential to the lockdowns and social restrictions. The cause of death with a persistently lower mortality is that of death from underlying diseases. This category was used to capture those children with an underlying condition able to explain their deaths, with most of them (69.0%) having an underlying chromosomal, genetic, or congenital anomaly identified as the main cause of death at their child death review. It remains likely that the precipitant or contributory factor in many of these cases may also have been an infectious agent in a child with complex needs. We hypothesize that better practices in hand washing and overall reduction in transmission of infections in this complex group may have continued benefits. However, while the overall association was that of a return to prepandemic levels, the pattern of mortality may have changed, with some impacts or temporal changes, persisting. The reductions seen during the pandemic, were similar across age groups, regions of England, sex, and socioeconomic deprivation. It is important to note that reductions in mortality were seen in all levels of deprivation and roughly equated to children experiencing a risk of death similar to that for children living in areas that were 2 deciles less deprived the previous year. This provides evidence that community actions can reduce the social-economic patterning of childhood mortality in England.^15^ Indeed, broad policy responses, enacting initiatives to provide cross-societal support may have helped this levelling up, and the impacts of returning to the status quo need to be watched carefully.^16,17^ However, the impact may have been more apparent in rural communities; although again, like the overall impact on infection, there was little difference to be seen in the following year. Again, this work is investigating the overall impact of the pandemic, not just deaths caused by COVID-19 itself, and it would seem reasonable that the measures put in place to restrict spread may have differential impacts across different environments. More space between households and a physically greater ability to socially isolate may underpin this finding; and further work may help identify generic mechanisms for further study.

Death by infection was also the clearest area to show variation of impact across different ages, with reductions in mortality in the first year of the pandemic potentially restricted to children between ages 1 and 15 years. The lack of a winter increase in deaths during 2020 to 2021, which was seen in the preceding and subsequent years, may reflect reductions in circulating infectious agents and may explain many of the findings here. This would be consistent with the reduction in mortality seen in ages 1 to 4 years (where direct deaths from COVID-19 are rare, but other infectious agents relatively common) and increases in mortality in the subsequent year for the oldest children. This appears consistent with the differential case fatality rate by age, with older people having greater risk, but it is striking in an age group with such low inherent mortality.

Underlying trends may also be playing a role in this work. The increasing trends in suicide are consistent with patterns seen in other countries,^18^ and ongoing scrutiny, and further targeted work in this area is needed to identify the most at-risk groups and where interventions may help. With preterm deaths being the biggest single cause of death in childhood, the lack of any measurable changes, despite broad effects of COVID-19 during pregnancy, health care changes, and staff impacts, appears striking. This may have been confounded by modest reduction in births and preterm deliveries in 2020.^19^ Alternatively, the effect of the pandemic may not have been considerable enough to be identified in a population with an already high risk of death.^20^

Finally, the changes observed in the numbers of SUDIC are complex, with no significant change from 2020 to 2021 compared with 2019 to 2020, and a slight increase during 2021 to 2022. SUDIC includes all unexpected deaths during childhood, many of which will be explained by an underlying disease process. This is the first national study to identify all unexpected childhood deaths. Further analysis of underlying causes and contributory factors will be reported separately, but this may be the first mortality signal we are seeing from families struggling with increasing poverty as we move out of the pandemic.

### Limitations

This study had limitations. This work is based on statutory data reported to NCMD, and previous work has shown good validation and coverage.^14^ However, we had some missing data on the demographics (eg, ethnicity), which should be considered when interpreting this work. A further limitation was our measure of the population at risk, which was derived from ONS data. However, numbers of deaths are small for most domains, and changes in the denominators are unlikely to make a difference in interpretation. The main limitation of this work is likely to be the precision of the estimates, with childhood death a rare event. While we were able to detect large changes in the relative risk of death between years and subgroups, small but important changes for particular groups may still have occurred but be difficult to interpret. One particular group is that of childhood suicides, which, while rare, are an important cause of (potentially avoidable) death in older children; but confidence intervals were wide and interpretation was difficult. In addition, the category of death in this work is provisional, based on information available at 48 hours, and further CDOP investigations are likely to modify this in some cases.

## Conclusions

In this cohort study of overall child deaths in England, the first year of the COVID-19 pandemic (ie, 2020-2021) showed a substantial reduction in all-cause child mortality, which returned to close to prepandemic levels the following year. However, there was still a net reduction in deaths despite this, with 4% fewer deaths over the 3-year period than would have been expected. Reductions in child deaths during the pandemic were seen across much of the population, notably in reductions of deaths from infection and underlying conditions, with reductions most noticeable in rural areas. However, the risk of death from trauma and infection in the oldest children has increased for each year analyzed in this study.

## Notes

Supplement 1. **eTable.** Causes of Child Deaths Reported to NCMD in England Between April 2019 and March 2022

Supplement 2.
Data Sharing Statement
