# Supplementary material for: Child Mortality in England During the First 2 Years of the COVID-19 Pandemic
Source: JAMA Netw Open. 2023 Jan 9;6(1):e2249191. doi: 10.1001/jamanetworkopen.2022.49191 (PMC9857017; doi:10.1001/jamanetworkopen.2022.49191)
Supplement: Supplement 1. — eTable. Causes of Child Deaths Reported to NCMD in England Between April 2019 and March 2022 [file jamanetwopen-e2249191-s001.pdf]

Supplemental Online Content

Odd D, Stoianova S, Williams T, Fleming P, Luyt K. Child mortality in England during the first 2 years of the COVID-19 pandemic. *JAMA Netw Open*. 2023;6(1):e2249191. doi:10.1001/jamanetworkopen.2022.49191

eTable. Causes of Child Deaths Reported to NCMD in England Between April 2019 and March 2022

This supplemental material has been provided by the authors to give readers additional information about their work.

© 2023 Odd D et al. *JAMA Network Open.*

eTable. Causes of Child Deaths Reported to NCMD in England Between April 2019 and March 2022

**Measure**	**N**	**Child deaths reported – Number (Percent)**			***P* value**
		**2019/2020**	**2020/2021**	**2021/2022**	
All Deaths	9872	3409	3035	3428	
Death by Cause	9626				<.001
Malignancy	760	256 (7.8)	257 (8.7)	247 (7.3)	
Preterm Birth	2738	903 (27.4)	873 (29.6)	962 (28.4)	
Intrapartum Event	538	166 (5.0)	188 (6.4)	184 (5.4)	
Infection	408	165 (5.0)	82 (2.8)	161 (4.8)	
Trauma	572	162 (4.9)	195 (6.6)	215 (6.4)	
Substance Abuse	39	20 (0.6)	10 (0.3)	9 (0.3)	
Suicide	361	109 (3.3)	119 (4.0)	133 (3.9)	
SUDIC	1340	433 (13.2)	417 (14.2)	490 (14.5)	
Underlying Disease	2870	1080 (32.8)	807 (27.4)	983 (29.1)	

© 2023 Odd D et al. *JAMA Network Open.*
