# Supplementary material for: Child Mortality in England During the First 2 Years of the COVID-19 Pandemic
Source: JAMA Netw Open. 2023 Jan 9;6(1):e2249191. doi: 10.1001/jamanetworkopen.2022.49191 (PMC9857017; doi:10.1001/jamanetworkopen.2022.49191)
Supplement: Supplement 2. — Data Sharing Statement [file jamanetwopen-e2249191-s002.pdf]

Data Sharing Statement

Odd. Child Mortality in England During the First 2 Years of the COVID-19 Pandemic. *JAMA Netw Open*. Published January 09, 2023. doi:10.1001/jamanetworkopen.2022.49191

Data
Data available: No

Additional Information
Explanation for why data not available: Aggregate data may be available on request to the corresponding author, and subject to approval by HQIP.
